# Alkaline Extraction for Lead Determination in Different Types of Commercial Paints

**DOI:** 10.3390/mps2040084

**Published:** 2019-11-01

**Authors:** David Romero-Estévez, Gabriela S. Yánez-Jácome, Karina Simbaña-Farinango, Pamela Y Vélez-Terreros, Hugo Navarrete

**Affiliations:** Centro de Estudios Aplicados en Química, Pontificia Universidad Católica del Ecuador, CESAQ-PUCE, Ecuador, Av. 12 de Octubre 1076 y Roca, Quito, Pichincha 17012184, Ecuador; gsyanez@puce.edu.ec (G.S.Y.-J.); kjsimbanaf@puce.edu.ec (K.S.-F.); pameyvt@gmail.com (P.Y.V.-T.)

**Keywords:** alkaline extraction, flame atomic absorption, regulation, solvent-base, water-base

## Abstract

In 2017, the World Health Organization and the United Nations Environment Programme formed the Global Alliance to Eliminate Lead Paint. All alliance member countries have pledged to develop control regulations that include lead threshold limits. To improve regulations and demonstrate compliance of paint industry products, it is necessary to have adequate, locally applicable methodologies. In this sense, the main objective of this research was to validate the methodology of alkaline extraction for the quantification of lead in ten different types of Ecuadorian commercial paints using flame atomic absorption spectrophotometry. Two hundred and fifty samples from different paint industry products were analyzed, and the results were used to evaluate the method’s performance and robustness. It was determined that the method could be applied for lead concentrations above 100 mg·kg^−1^, and results showed relative standard deviation values lower than 14.8% and fortification recoveries between 80.3 and 119.4%, fulfilling the acceptance criteria established in the Environmental Protection Agency’s lead-based Paint Laboratory Operations Guidelines.

## 1. Introduction

Lead (Pb) is a toxic metal that can accumulate in living tissue and affect the neurological, cardiovascular, and renal systems [1,2]. Humans’ intake of Pb alters the normal physiology of their biological systems. Although it has not yet been demonstrated that dermal exposure is an important entry route for humans [3], in the case of children, hand-to-mouth behaviors and the rapid absorption of ingested Pb increase its health risks.

The main sources of Pb exposure are contaminated food and water intake. Additional means of exposure include contact with toys, paints, coatings, and metallic materials such as jewelry that contain this metal [4,5,6]. According to statistical studies conducted by the World Health Organization (WHO), Pb exposure accounted for approximately half a million deaths in 2016 [7], 82% of which occurred in developing countries [8]. Architectural paints and household items are an important exposure source because of their proximity to people; these products also continue to be sold despite their high Pb content [9]. In 2017, the United Nations Environment Programme (UNEP) reported that only one-third of 193 countries have regulations related to the use of Pb in paints [8]. In response to the frequent incidence of high Pb concentrations in paints, especially in decorative glazes, and the possible health conditions that they could cause, the WHO, together with the UNEP, formed the Global Alliance to Eliminate Lead Paint (GAELP) [9,10]. This alliance has as its main objective the elimination of Pb in paints by 2020 [8]. Every alliance member country has pledged to develop control regulations that include Pb threshold limits. 

To perform paint quality control and establish Pb threshold values, there are several analytical methods described in the literature. These methods include the following: dry ashing [11] and wet acid digestions either on a heating plate or in a microwave [12] to prepare samples; atomic absorption spectrophotometry [5,11,13,14,15]; inductively coupled plasma emission spectroscopy quantification [16,17,18,19,20]; the Delves micro-sampling technique [21]; and non-destructive techniques such as X-ray fluorescence [4] for quantification. In Ecuador, the most frequently used methods are the dry ashing technique [11,22] and wet acid digestion [22]. 

In its Pb-Based Paint Laboratory Operations Guidelines: Analysis of Pb in Paint, Dust, and Soil [12], the Environmental Protection Agency (EPA) does not recommend using the dry ashing technique because it is difficult to control. In addition, heating homogeneity cannot be guaranteed, and avoiding splatter/cross-contamination of samples is challenging. Wet acid digestion techniques are preferable to dry ashing, but when only nitric acid is used, an incomplete digestion process can occur. A different wet acid digestion technique that uses a mixture of nitric acid and hydrogen peroxide may be suitable for the digestion of most samples. Nevertheless, these methods have not been validated for certain matrices, including solvent-based paints, epoxy, alkyd enamels, and synthetic enamels, among others. Methods that use perchloric acid in combination with nitric acid have shown acceptable results, but they are not recommended because of the myriad of safety precautions for perchloric acid use. On the other hand, most of the methods described in the literature have been applied to dry paint products (paint chips or powder) [5,11,13,14,15]; therefore, the acceptable performance of these techniques for liquid products or solvent-based paints is still unknown.

An alternative less commonly used method known as Method A is described in ISO 6503:1984 [23]. This method consists of wet oxidation of the sample using a mixture of sulfuric acid and hydrogen peroxide, followed by an alkaline extraction of Pb in the residue with ethylenediaminetetra-acetic acid (EDTA) and ammonia solution. This ISO alternative method is meant to coat materials with Pb content in the range of about 0.01% mass fraction (*w*/*w*) 2% (*w*/*w*). The principle behind this extraction technique involves forming a Pb-chelating ligand using the EDTA as a chelating agent and ammonium cation ligands. This technique is commonly used for metal extraction from soil matrices [24,25,26,27].

In 2015, the Ecuadorian Standardization Service (abbreviated INEN in Spanish) formed the National Technical Committee for Paints (abbreviated CTN in Spanish). This committee discussed the inclusion of Pb threshold values in the standards for all paint products according to their intended use. This process is still ongoing, and the threshold values are expected to be included in the new versions of all paint standards and regulations. Presently, a threshold value of 100 mg·kg^−1^ of Pb has been established for architectural and domestic use paints [28,29] and a 600 mg·kg^−1^ threshold value for traffic and vehicular use paints [30]. Regarding standards for other paint products, like traffic paints and synthetic alkyd enamels for domestic use [31,32], the only requirement is that “the raw materials must be free of Pb”. Furthermore, for some paints and coatings, including anti-corrosive primers, anti-corrosive coatings, nitrocellulose and polyester putties, lacquers, and wood sealants [33,34,35,36,37,38,39,40,41,42,43,44], there are no established Pb regulations.

Because of the drafting of these new standards, there is now an imperative to develop local methodologies to ensure that industries are producing, importing, and marketing Pb-free paints. In response to this need, the main objective of this study was to validate the analytical ISO 6503:1984 alkaline extraction method [23] to quantify Pb in ten different types of paints. To accomplish this, different local producers and marketers provided paint samples; thus, this study constitutes the first effort to determine Pb content in local products in Ecuador.

## 2. Materials and Methods

The initial tests were carried out using trace metals—i.e., paint chips, a certified reference material (Sigma-Aldrich Inc; CRM013-50G)—and different methods including dry ashing [11,23], wet acid digestion [12], and alkaline extraction [23] techniques. The final analysis method selected for the samples was alkaline extraction, a wet digestion procedure known as Method A in ISO 6503:1986 [23]. This method was chosen because it complies with the quality assurance parameters concerning precision and accuracy established in the EPA’s Pb-Based Paint Laboratory Operations Guidelines [12].

Between April 2015 and January 2019, 10 main local manufacturers and marketers provided 250 paint samples corresponding to water-based paint, solvent-based paint, epoxy, alkyd enamels, synthetic enamels, lacquers, putties, pure pigments, preformed thermoplastics, and resins. All the samples were analyzed using the alkaline extraction and flame atomic absorption spectrophotometry (FAAS) methodology [23].

**Figure 1:**
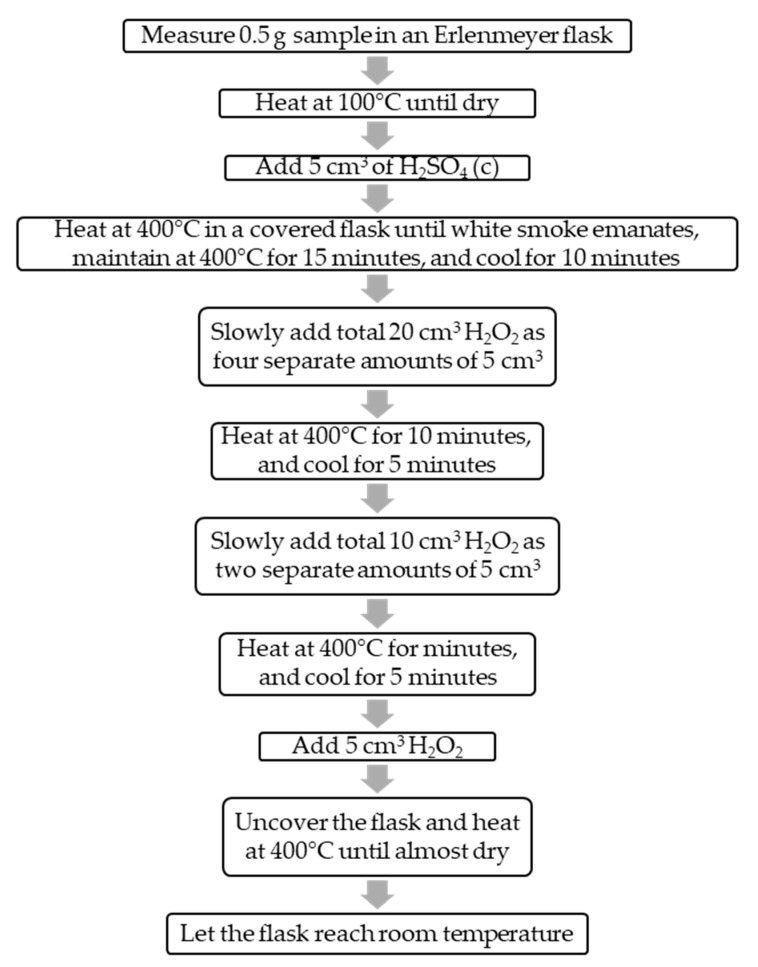
Paint decomposition process. Erlenmeyer flasks were used instead of glasses [23] to avoid splatter.

The paint decomposition process was done according to the procedure shown in Figure 1 using a heating plate (OVAN, model MMH90E), sulfuric acid (Pharmco-Aaper, analytical grade CAS# 7664-93-9), and 30% hydrogen peroxide (Fisher Chemical, analytical grade CAS# 7722-84-1).

**Figure 2:**
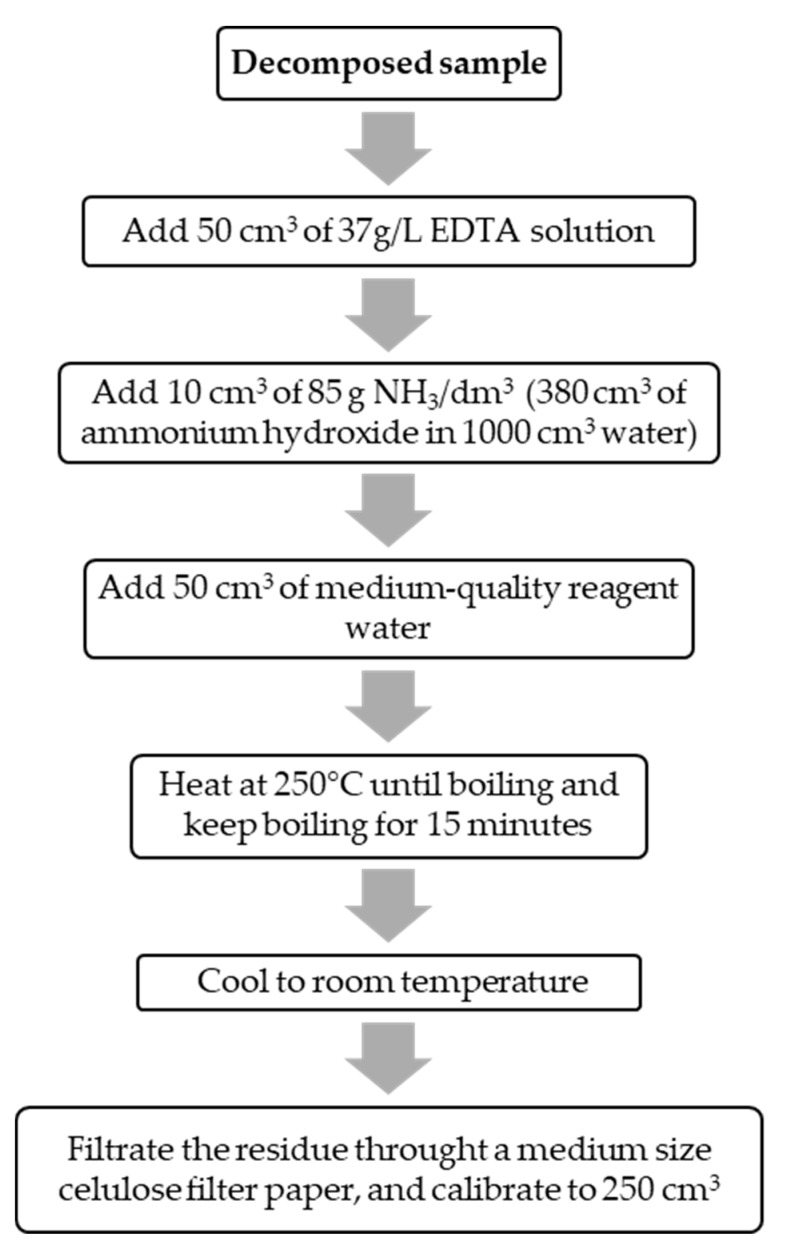
Lead alkaline extraction from the paints.

The Pb extraction process from paints was done according to the procedure shown in Figure 2 using disodium salt of EDTA (Lobachemie, analytical grade, CAS# 6381-92-6), an ammonium hydroxide solution (Merck Trademark 25%, analytical grade, CAS# 1336-21-6), and medium-quality reagent water.

The Pb quantification was conducted using FAAS (Perkin Elmer, AAnalyst 400) with a hollow Pb cathode lamp (Perkin Elmer). Calibration curves were performed with 0.2, 1.2, 3.0, and 5.0 mg·dm^−3^ standard dilutions prepared from a 1000 mg·dm^−3^ certified reference material (Inorganic Ventures, catalog CGPB1). Linearity was evaluated with regression analysis, where a lack-of-fit test was used at a 95% confidence level for the daily group of results of all the external standards used (i.e., samples fortifications, calibration curve control standards).

The limits of detection (LOD) for Pb were based on the variability of the blank, calculated by multiplying the standard deviation of the mean blank concentration values by three [12]. The limits of quantification (LOQ) values were determined experimentally by analyzing fortification samples in low concentrations that fall within the EPA precision and accuracy acceptance criteria [12].

Quality assurance was carried out following the specifications described in Section 3.4.1 of the EPA guidelines [12]. Precision was determined using triplicates of each sample analysis and expressed as the relative standard deviation (RSD). Accuracy was calculated using fortification recoveries by adding 150 mg·kg^−1^ of Pb to each sample, since specific product reference materials for each type of paint do not exist. Fortifications were simultaneously analyzed with the samples, and expressed as recovery rates. In addition, initial calibration verification, an initial calibration blank, continuing calibration verification, and method blank verification were done for quality assurance. 

The acceptance criteria used was in accordance with Section 3.4.4 of the EPA guidelines [12], as follows:

- Initial calibration verification: within ±10% of the known value of the standard control.
- Initial calibration blank: an absolute value of no more than 20% of the LOQ value.
- Continuing calibration verification: within ±10% of the known value of the standard control.
- Continuing calibration blank: an absolute value of no more than 20% of the LOQ value.
- Matrix spike (fortifications): within ±25% of the known value.
- Triplicate sample: within ±25% of the RSD.
- Method blank: an absolute value of no more than 20% of the LOQ value.

## 3. Results

### 3.1. Method Validation

The initial tests were carried out using dry ashing and wet acid digestion techniques [11,15,22], without obtaining satisfactory precision and accuracy results. In several cases, RSD results were higher than 25% and fortification recoveries lower than 75%. For this reason, the alkaline extraction process was selected as an adequate preparation technique, since its performance was in accordance with the acceptance criteria described by the EPA [12] in Section 3.4.4: RSD values lower than 25% and fortifications recoveries within 100 ± 25%.

During the validation process, for each day of analysis, a 0.2 mg·dm^−3^ standard was analyzed in terms of initial and continuing calibration verification; recovery results obtained were within 100 ± 10% (internal laboratory acceptance criteria). All the calibration blanks (initial, continuing, and method) were lower than 0.009 mg·dm^−3^ (4.5% of the LOQ value). 

Each sample was analyzed in triplicate to verify precision; in addition, a fortification was used for each sample to ensure the accuracy of the extraction method performed. 

As seen in the results shown in Table 1, the alkaline extraction methodology with FAAS was adequate to quantify Pb concentrations between 100 and 21,044.5 mg·kg^−1^. Instrumental LOD was calculated using the RSD for the blanks (multiplied by three); the LOD value was 0.5 mg·kg^−1^. Although the LOQ was obtained using fortifications in low concentrations; the LOQ was 100 mg·kg^−1^, and this value was not only within the acceptance criteria of the EPA Pb-Based Paint Laboratory Operations Guidelines [12], but it was also adequate to evaluate the Pb content using the Ecuadorian regulations. The maximum RSD obtained for all types of paints was 14.8%; this value was lower than the EPA acceptance criteria (25%). Considering all the types of paints analyzed, the fortification recoveries were between 80.3 and 119.4%, which were within the acceptance criteria recoveries corresponding to between 75 and 125%.

### 3.2. Pb Content of Paint Samples

The sample results varied (Table 1); some samples reached values up to 21,044.5 mg·kg^−1^ (average 5578.5 mg·kg^−1^). On the other hand, there were samples with results lower than the LOD. The samples below the LOD were water-based paints (4.4%), solvent-based paints (5.3%), synthetic enamels (2.5%), mastics (20.0%), pigments (22.2%), and thermoplastic preforms (37.5%). 

## 4. Discussion

The dry ashing techniques [11,22] showed fortification recoveries lower than 75% (data not shown). There were two presumed reasons for this low performance: first, dry ashing techniques are difficult to control, and there is a possibility of uneven heating or splatter/cross-contamination. Second, the exclusive use of nitric acid for the ash digestion could result in an incomplete digestion process [12].

The wet acid digestion technique [23] was only applied on pigmented coatings to evaluate the calcination method; nevertheless, the American Society for Testing and Materials ASTM [11] mention that “there is no reason to believe that varnishes and lacquers could not be analyzed successfully, provided that appropriate precautions are taken”. 

When alternative hot plate digestion techniques [15] were used, not all cases resulted in acceptable fortification recoveries. The EPA mentions that wet digestion techniques that use a mixture of nitric acid and hydrogen peroxide can be used for Pb extraction from most types of paint products. However, a complete method validation is necessary for each type of paint product to ensure its performance [12]. The use of sulfuric acid instead of nitric acid could help guarantee a complete digestion process for most paint components, also allow complete oxidation of organic matter, breaking the organic-metal ligands.

In the case of microwave digestion techniques, the Truman State University experimental procedure [15] has only been tested with paint chips, which does not guarantee its acceptable performance for liquid paint products. In addition, the danger of applying high temperatures and pressures to solvent-based paints, lacquers, and other products that contain explosive or flammable compounds must be considered.

The validation results confirmed the adequate performance of the alkaline extraction methodology and the Pb quantification using FAAS. Regarding the quality assurance of the results, the recovery results measured against the calibration curve control standards (initial and continuing) were below 10%, and all the calibration blanks (initial, continuing, and method) were lower than 20% of the LOQ value, which was within the acceptable range established in Section 3.4.4 of the EPA’s guidelines [12]. The triplicate RSD values were below 25%, and the recovery rates of the fortifications were between 75 and 125%. The fulfillment of all the aforementioned criteria shows the adequate performance of the analytical method, and its applicability for the 10 types of paint products included in the study. The robustness of the methodology was evaluated by comparing the results obtained for every type of paint product to the EPA acceptance criteria [12].

The sample results demonstrated not only the varied Pb content among and within the different types of paints but also that, in some cases, the Pb concentrations were above the threshold values stipulated in the Ecuadorian regulations. In the case of synthetic enamels (average 5578.5 mg·kg^−1^), solvent-based paints (average 1316.7 mg·kg^−1^), and epoxy paints (average 167.8 mg·kg^−1^), the Pb content exceeded the threshold values by 20 and 50 times for paints for traffic and architectural use, respectively.

In a related study conducted by Clark et al. [9] in several different countries, products with Pb contents of 25,000 mg·kg^−1^ (Armenia), 15,700 mg·kg^−1^ (Kazakhstan), 16,600 mg·kg^−1^ (India), and 5600 mg·kg^−1^ (Brazil) were identified. In Brazil, implemented regulations had led to the reduction of the Pb content as the products initially contained an average of 36,000 mg·kg^−1^ of Pb. All these results show similar values to those of the present study (the highest Pb concentration was 21,044.5 mg·kg^−1^). In another study conducted by Gottesfeld, Pokhrel, and Pokhrel, [5] on decorative paints in Nepal, the Pb concentration values were between 3448.0 and 54,755.0 mg·kg^−1^ with an average concentration of 5100 mg·kg^−1^. These values were higher than the results in the present study.

**Figure 3:**
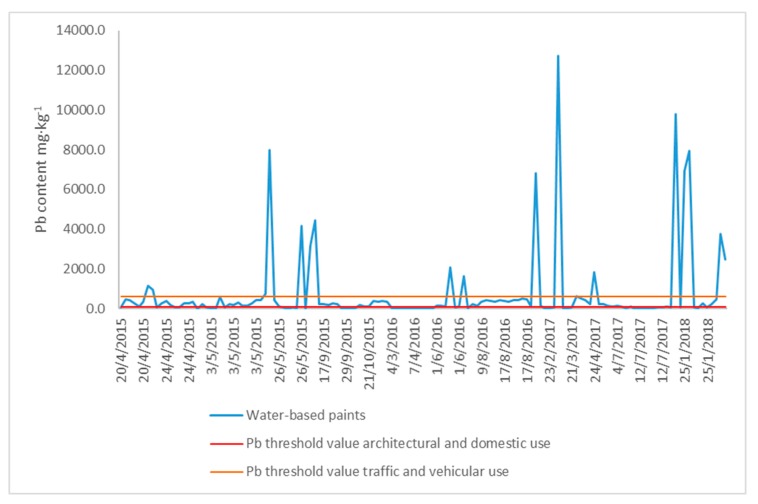
Lead content (mg·kg^−1^) in water-based paint samples analyzed between April 2015 and February 2018.

In 2015, after the CTN discussed the availability of an analytical method that would allow for the quantification of Pb content in paint products, the principal aim was not only to validate the methodology but also to begin improving the production processes for Pb-free paints. However, according to the water-based paint results presented in Figure 3, no significant improvement or reduction of Pb content was observed. This situation is worrisome since, in 2019, the Pb threshold values came into force, and both manufacturers and marketing companies are aware that they are now active. Additionally, control processes have to be implemented to determine compliance with the regulations in question.

## 5. Conclusions

The results of the current study show that the alkaline extraction method was satisfactory for determining Pb content in 10 types of paints and coatings: water-based paint, solvent-based paint, epoxy, alkyd enamels, synthetic enamels, lacquers, putties, pure pigments, preformed thermoplastics, and resins. The methodology was validated for quantifying Pb from concentrations up to 100 mg·kg^−1^, which corresponds to the Ecuadorian regulations’ [28,29] lowest threshold value, to the highest concentration analyzed of 21,044.5 mg·kg^−1^. 

The dry ashing and wet acid digestion techniques have some limitations, and it is difficult to have a complete control over them. It is also hard to guarantee the complete digestion or extraction of the Pb content. On the other hand, techniques such as inductively coupled plasma emission spectroscopy quantification and X-ray fluorescence are expensive. Thus, it is necessary to have reliable and relatively low-cost alternative methods available to control Pb content in paint products that are manufactured or marketed both nationwide and worldwide.

The results of the water-based samples analyzed between April 2015 and February 2018 showed that, even when some threshold limit standards exist, there still are paint products with a high content of Pb. This study was the first to quantify Pb content in different types of paint products with a relatively easy technique, in accordance with the Ecuadorian governmental authorities, responsible for controlling toxic metal content in different commercial products.

## Figures and Tables

**Table 1:** Lead content range (mg·kg^−1^), rates of standard deviation (RSD, %), and fortification recovery rates (%).

Paint Product	Number of Samples	Pb Concentrations			RSD			Fortification Recovery Rates		
		Minimum Value	Maximum Value	Mean Value	Minimum Value	Maximum Value	Mean Value	Minimum Value	Maximum Value	Mean Value
water-based ^1^	135	< LOD	12,715.3	738.4	0.0%	14.8%	4.7%	80.8%	119.2%	101.1%
solvent-based	38	< LOD	10,502.9	1316.7	0.0%	13.7%	4.7%	80.4%	119.4%	101.4%
epoxy	5	16.2	3081.7	1167.8	0.7%	11.6%	4.7%	83.9%	114.1%	100.2%
alkyd enamels ^2, 3^	4	172.8	727.7	447.0	0.9%	8.1%	5.0%	82.2%	108.7%	97.0%
synthetic enamels	40	< LOD	21,044.5	5578.5	0.0%	13.4%	2.8%	80.3%	119.3%	101.7%
lacquers ^1^	2	483.4	826.5	654.9	4.7%	6.0%	5.3%	94.0%	109.0%	101.5%
putties	5	< LOD	98.5	37.1	0.0%	12.4%	5.6%	94.7%	109.3%	103.4%
pure pigments	9	< LOD	145.1	33.1	0.0%	11.4%	3.7%	92.0%	118.4%	101.4%
preformed thermoplastics ^2^	8	< LOD	77.0	20.9	0.0%	2.7%	1.6%	86.6%	107.1%	97.9%
resins	4	4.6	189.9	124.4	0.8%	10.8%	7.4%	102.0%	115.9%	107.4%
TOTAL	250	4.6	21,044.5	1011.9	0.0%	14.8%	4.6%	80.3%	119.4%	101.3%

LOD: Limits of detection.

Ecuadorian regulations’ requirement specifications:

^1^ Architectural and domestic use paints: threshold value 100 mg·kg^−1^ [28,29].

^2^ Raw materials must be Pb-free [30].

^3^ Traffic and automotive industry paints: threshold value 600 mg·kg^−1^ [31,32].
